# An Unusual Genetic Variant of Long QT Syndrome with Late Presentation in the Sixth Decade

**DOI:** 10.19102/icrm.2026.17064

**Published:** 2026-06-15

**Authors:** Arindam Pande, Sanjeev S. Mukherjee, Ashesh Halder

**Affiliations:** 1 Department of Cardiology, Manipal EM Bypass Hospital, Kolkata, India; 2 Department of Cardiology, AIIMS, Kalyani, India

**Keywords:** Automated ICD, long QT syndrome, polymorphic ventricular tachycardia, syncope, torsades de pointes.

## Abstract

This case report describes a 57-year-old woman with a history of transient loss of consciousness, initially treated as epilepsy, who presented with recurrent torsades de pointes requiring direct-current cardioversion. Her evaluation revealed a prolonged corrected QT (QTc) interval, and, after excluding acquired causes, she was managed with a dual-chamber implantable cardioverter-defibrillator, β-blocker and mexiletine therapy, and a base rate of 80 bpm. Genetic analysis identified a previously unreported, possibly pathogenic compound heterozygosity in the *AKAP9* gene (c.9443C>T, p.Thr3148Met and c.10515_10520delAACCGG, p.Thr3506_Gly3507del). Upon diagnosis of congenital long QT syndrome, her antiepileptic drugs were discontinued. At 6-month follow-up, she remained free of arrhythmic events with noted improvement in her QTc interval, highlighting the critical importance of accurate diagnosis and genotype-guided therapy in such cases.

## Case presentation

A 57-year-old woman presented with a 2-month history of recurrent, transient loss of consciousness. Each episode lasted 30–60 s and was preceded by giddiness; one event resulted in a fall from a sitting to a bed position. There were no associated symptoms of bowel or bladder incontinence or post-event neurological deficits. Her medical history was significant for mild right-sided sensorineural hearing loss, and she reported the first similar episode 22 years prior, with a marked increase in frequency over the past decade. Prior medical records indicated a previous hospitalization for involuntary limb movements, leading to a neurological assessment and a subsequent long-term diagnosis of epilepsy treated with regular antiepileptic medication. There was no personal history of coronary artery disease (CAD) and no family history of premature CAD or sudden cardiac death.

**Figure 1:**
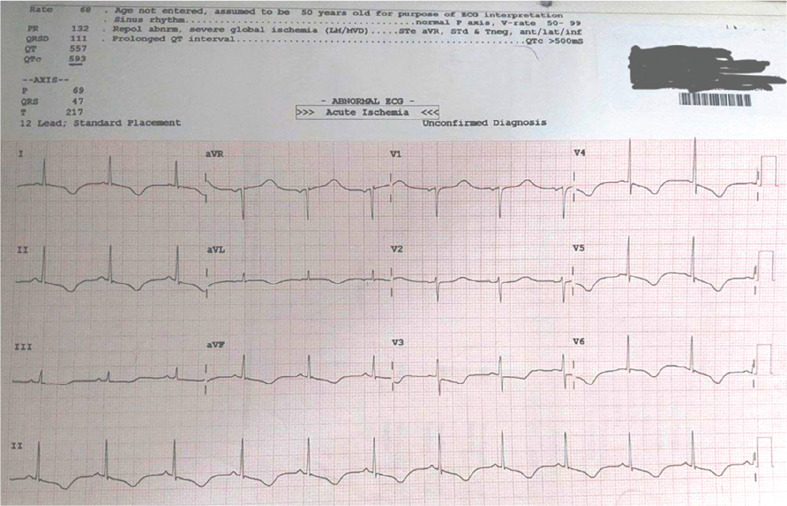
Electrocardiogram on admission showing long corrected QT interval at rest.

Her clinical evaluation did not reveal any abnormality, and her resting electrocardiogram (ECG) is shown in **Figure 1**.

**Figure 2:**
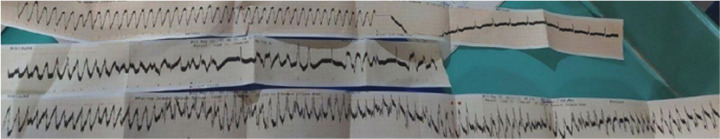
Loss of consciousness due to torsades de pointes.

Her blood chemistry demonstrated normal electrolytes and renal functions, and transthoracic echocardiography revealed normal left ventricular systolic function with normal epicardial coronaries on angiography. Her 24-h Holter monitoring in the outpatient clinic showed multiple runs of non-sustained ventricular tachycardia. She had also undergone cardiac magnetic resonance imaging in the past, which did not reveal any structural abnormality or scar. During her inpatient stay, she experienced a similar episode, and her ECG captured ventricular arrythmia that required cardioversion, as shown in **Figure 2**.

She underwent implantation of a dual-chamber implantable cardioverter-defibrillator (ICD), and the baseline rate was set at 90 bpm, with maximum tolerated β-blocker and mexiletine doses. We considered the possible next course of action: (1) discharge her on β-blocker therapy, (2) order ECG on first-degree relatives, or (3) order genetic testing.

Written informed consent was obtained from the patient for the publication of this case report and accompanying images.

## Discussion

This patient has possible long QT syndrome (LQTS), but this revelation was not sufficient for discharge owing to the caveats raised by her age, late presentation, and unusually malignant episodes. Genetic testing can also help in risk stratification based on information about the genotype, gender, QT duration, and history of cardiac events.^1^

The ECGs of her surviving brother and other first-degree relatives did not show a prolonged QT interval. Thus, the third suggested option—ordering genetic testing—was considered the most acceptable approach. We know that acquired LQTS is associated with QT prolongation and torsades de pointes (TdP) triggered by hypokalemia; bradycardia; and certain drugs such as macrolide antibiotics, fluoroquinolones, and antihistamines such as terfenadine. In this case, her corrected QT (QTc) interval was always around 570–590 ms, and she refused being on any such medications. Congenital LQTS is a cardiac ion channelopathy affecting myocardial repolarization typified by a prolonged QT interval on an ECG. Patients with prolonged QT intervals are at a high risk of syncope and sudden cardiac death due to polymorphic ventricular tachycardia. Many mutations in the potassium channel have been linked to LQTS. These include mutations in the *KCNQ1* (LQT1) and *hERG* (LQT2) genes as well as mutations in the sodium channel *SCN5A* (LQT3) gene. Female sex is considered to be an independent risk factor for TdP. ICD and β-blocker therapy usually suffice, but, rarely, surgical left cardiac sympathetic denervation is required to reduce the QTc interval and cardiac events.^2^

Her blood sample was sent for genetic analysis, where it underwent next-generation sequencing and subsequent mutation confirmation with traditional capillary Sanger sequencing analysis. The report indicated a possible causative non-synonymous mutation in the DNA: heterozygosity for A-kinase–anchoring protein 9 (AKAP9) exon 39 c.9443C>T (p.Thr3148Met) and exon 42 c.10515_10520delAACCGG (p.Thr3506_Gly3507del) mutation **(Table 1)**.

**Figure 3:**
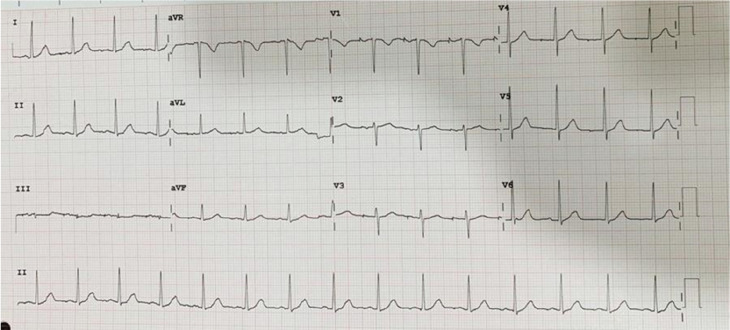
Atrial-based pacing at 90 bpm shows a corrected QT interval of 421 ms.

There are not many case reports of this variant in the literature, and it has not been previously described in the Human Gene Mutation Database. No other possible causative mutations have been identified. In the literature, the *AKAP9* gene is described as a modifier of LQTS clinical phenotype by altering QTc duration and influencing the risk of cardiac events and the severity of the disease. In particular, variants that impair the function or expression of the AKAP9-encoded Yotiao gene are prone to influence channel properties, explaining the phenotypic differences described in the literature.^3,4^ From this perspective, our case suggests possible association between the novel mutation found and potential life-threatening complications. She was discharged on 50 mg of metoprolol twice daily and 150 mg of mexiletine twice daily. She has done well during a short-term follow-up of 6 months, without experiencing any further episodes of TdP. Her device logs were reconfirmed, and an ECG was recorded **(Figure 3)** with a QTc interval of 421 ms.

We now know that *AKAP9* is a protein-coding gene located in the long (q) arm of chromosome 7 at position 21.2 (cytogenetic location: 7q21.2). AKAPs are a group of scaffolding proteins that have the common function of binding to the regulatory subunit of protein kinase A (PKA) determining the localization of PKA and enzymes that regulate the PKA pathway, such as phosphatases or phosphodiesterases, and other kinases, such as protein kinase C and protein kinase D. In the heart, AKAP-mediated macromolecular complexes coordinate three critical ion channel proteins: the ryanodine receptor, or intracellular calcium release channel, the L-type calcium channel, and the slowly activating delayed rectifier IK potassium channel. De Villiers et al. studied members of a South African LQTS-type 1 founder population (181 noncarriers and 168 mutation carriers) carrying the identical-by-descent *KCNQ1* p.Ala341Val (A341V) mutation and evaluated the modifying effects of *AKAP9* variants on heart rate–QTc interval, cardiac events, and disease severity.^4^ They noted a definite effect on QTc interval, cardiac event risk, and disease severity in mutation carriers, but it was not uniformly predictable.

Our patient has a different mutation, which has not been reported before. In view of the severely symptomatic nature and frequent TdP, which showed increase in frequency with age, we must study a larger population to define this specific variant and its phenotypic expression.

When faced with the diagnosis of congenital LQTS in adults, it is important to undertake special risk stratification. International LQTS Registry data demonstrate that the severity of LQTS in adulthood can be risk-stratified based on information about the genotype, sex, QT duration, and history of cardiac events.^1^

Genetic variation can increase susceptibility to acquired QT interval prolongation.^5^ The heritability of QT interval duration in the general population (excluding congenital LQTS patients) is about 35%.^6^ An important fact to consider is that first-degree relatives of patients with congenital LQTS can be at a higher risk of drug-induced QT prolongation as compared to nonrelated people.^7^ Several genes associated with a prolonged QT interval have been identified, such as the nitric oxide synthase 1 adaptor protein gene (*NOS1AP*), located on chromosome 1,^8^ which inhibits the L-type calcium channel. This gene has an impact on impulse propagation. Other gene mutations include polymorphisms within mutated genes in congenital LQTS and genes associated with intracellular calcium handling.^9^

## Conclusion

LQTS is among the most extensively characterized malignant channelopathies. When a prolonged QTc interval persists despite the elimination of acquired triggers, an underlying genetic etiology should be investigated. First-line therapy consists of pharmacotherapy with β-blockers; however, a subset of nonresponders may require ICD placement for secondary prevention. Advances in genetic research are increasingly enabling the identification of pathogenic mutations in early life, which can significantly alter disease prognosis. This evolving capability is critical for developing optimal, personalized management strategies for affected patients.

**Table 1:** Genetic Mutation Report Showing the *AKAP9* Mutation and Its Location

Gene and Transcript	Variant	Location	Zygosity	Disorder (OMIM)	Inheritance	Classification
AKAP9 NM_005751.5	c.9443C>T (p.Thr3148Met)	Exon 39	Heterozygous	Long QT syndrome 11 (611820)	Autosomal dominant	Uncertain significance
AKAP9 NM_005751.5	c.10515_10520delAACCGG (p.Thr3506_Gly3507del)	Exon 42	Heterozygous	Long QT syndrome 11 (611820)	Autosomal dominant	Uncertain significance

*Abbreviation:* OMIM, Online Mendelian Inheritance in Man.
